# miR-1, miR-133a, miR-29b and skeletal muscle fibrosis in chronic limb-threatening ischaemia

**DOI:** 10.1038/s41598-024-76415-9

**Published:** 2024-11-26

**Authors:** Alan J. Keane, Clara Sanz-Nogués, Dulan Jayasooriya, Michael Creane, Xizhe Chen, Caomhán J. Lyons, Isha Sikri, Katarzyna Goljanek-Whysall, Timothy O’Brien

**Affiliations:** 1 Regenerative Medicine Institute (REMEDI), University of Galway, Biomedical Sciences 1st Floor South, Corrib Village, Dangan, Galway, Ireland; 2 CÚRAM SFI Research Centre for Medical Devices, University of Galway, Galway, Ireland; 3 Institute of Life Course and Medical Sciences, University of Liverpool, Liverpool, UK

**Keywords:** Chronic limb-threatening ischaemia, Fibrosis, Muscle regeneration, MicroRNAs, Preclinical research, Translational research

## Abstract

Chronic limb-threatening ischaemia (CLTI), the most severe manifestation of peripheral arterial disease (PAD), is associated with a poor prognosis and high amputation rates. Despite novel therapeutic approaches being investigated, no significant clinical benefits have been observed yet. Understanding the molecular pathways of skeletal muscle dysfunction in CLTI is crucial for designing successful treatments. This study aimed to identify miRNAs dysregulated in muscle biopsies from PAD cohorts. Using *MI*croRNA *EN*richment *TUR*ned *NET*work (MIENTURNET) on a publicly accessible RNA-sequencing dataset of PAD cohorts, we identified a list of miRNAs that were over-represented among the upregulated differentially expressed genes (DEGs) in CLTI. Next, we validated the altered expression of these miRNAs and their targets in mice with hindlimb ischaemia (HLI). Our results showed a significant downregulation of miR-1, miR-133a, and miR-29b levels in the ischaemic limbs versus the contralateral non-ischaemic limb. A miRNA target protein-protein interaction network identified extracellular matrix components, including collagen-1a1, -3a1, and -4a1, fibronectin-1, fibrin-1, matrix metalloproteinase-2 and -14, and Sparc, which were upregulated in the ischaemic muscle of mice. This is the first study to identify miR-1, miR-133a, and miR-29b as potential contributors to fibrosis and vascular pathology in CLTI muscle, which supports their potential as novel therapeutic agents for this condition.

**Supplementary Information:**

The online version contains supplementary material available at 10.1038/s41598-024-76415-9.

## Introduction

Peripheral arterial disease (PAD) most often occurs due to atherosclerotic disease in the peripheral arteries, resulting in skeletal muscle ischaemia distal to the point of vessel narrowing^1^. Most patients with PAD are asymptomatic. However, impaired perfusion of the lower limbs is associated with muscle atrophy, functional decline and increased risk of morbidity and mortality^2–5^, even in those patients without obvious symptoms^6^. Patients with PAD can exhibit intermittent claudication (IC), defined as limb pain during physical activity which resolves at rest^7^. This may occur when arterial narrowing is such that limb perfusion is insufficient to meet muscle metabolic demands during exercise. It is estimated that up to 10% of patients with PAD progress or present de novo with chronic limb-threatening ischaemia (CLTI), the most severe manifestation of PAD^8^. CLTI is characterised by chronic ischaemic rest pain, non-healing ulcers, and gangrene and is associated with a high risk of limb loss, cardiovascular morbidity and mortality^7^. Interestingly, most patients with IC do not naturally progress to CLTI^9–11^, suggesting that these two manifestations of PAD may represent distinct pathogenic stages of the disease despite similar ischaemic aetiology.

Current bioinformatics and omics-based approaches have provided important insights into the pathophysiology of clinical PAD. Ryan et al. recently reported a unique skeletal muscle mitochondriopathy that distinguished patients with CLTI from those with IC^12^. Other authors have utilised this publicly available RNA-sequencing dataset of PAD cohorts to elucidate the pathological signatures that discriminated CLTI from IC. For instance, Cong et al. identified fibrosis pathways involving transforming growth factor-β (TGF-β), collagen deposition, and vascular endothelial growth factor (VEGF) signalling as a novel gene expression feature for CLTI but not IC^13^. Yao et al. identified several genes encoding subunits of the human complement system as promising markers for discriminating between CLTI and IC^14^. More recently, Ferrucci et al. multi-omics analysis using RNA sequencing and proteomics on PAD skeletal muscle (free from diabetes mellitus (DM) and CLTI) reported that mitochondrial dysfunction and fibrotic are processes active in PAD without CLTI^15^. These results contradict to some extent the findings from Ryan and Cong et al. In another study using a different transcriptomic dataset, Saini et al. reported differential gene and microRNA (miRNA) expression associated with PAD progression^16^.

miRNAs are small non-coding RNAs ~ 22 nucleotides in length which regulate gene expression at the post-transcriptional level^17^ and are considered robust regulators of muscle development and homeostasis^18^. miRNAs have been thoroughly investigated in the context of PAD^19,20^, in particular, as potential biomarkers in skeletal muscle^21,22^ and circulation^23–26^. However, the full spectrum of dysregulated miRNAs associated with PAD stages is poorly understood. This study aimed to identify dysregulated miRNAs in gastrocnemius muscle biopsies that were differentially expressed in different PAD cohorts. We analysed a publicly available RNA-sequencing database of PAD cohorts^12^ using *MI*croRNA *EN*richment *TUR*ned *NET*work (MIENTURNET), a web tool for miRNA target enrichment and network-based analysis^27^. We hypothesised that transcriptomic analysis using this bioinformatics tool will identify CLTI-specific dysregulated miRNAs and their associated targets. Subsequently, we validated the findings in hindlimb ischaemia (HLI) mice, a preclinical model of CLTI^28,29^.

## Methods

### Data acquisition

RNA-sequencing-based profiles from CLTI, IC and non-PAD adults were acquired from a dataset published by Ryan and colleagues^12^. In Ryan et al. study, gastrocnemius muscle biopsies were obtained from patients with IC (*n* = 27), CLTI (*n* = 19) and non-PAD controls (*n* = 32) for whole transcriptome sequencing. RNA sequencing was performed by sequencing paired-end (150 bp) reads on an Illumina HiSeq 4000. The authors identified differentially expressed genes (DEGs) in skeletal muscle from CLTI vs. non-PAD control, CLTI vs. IC and IC vs. non-PAD controls using edgeR with a p-value < 0.05 and a fold change > 1.5^12^. The diagnostic criteria, participant demographics, and further methodological detail are described in Ryan et al. study.

### miRNA-target interaction enrichment analysis

DEGs in CLTI vs. non-PAD controls, CLTI vs. IC and IC vs. non-PAD controls were analysed using the online bioinformatics tool MIENTURNET^27^ (Accessed from Sep 2021 to Dec 2023). Upregulated and downregulated DEGs were analysed separately. DEGs were input to the MIENTURENT tool and miRNA-Target Interaction (MTI) enrichment was performed based on validated MTIs in the miRTarBase database. Results were filtered by setting a threshold for the minimum number of MTIs as 2 and FDR < 0.05. The enriched MTIs were visualised using Cytoscape^30^.

### Protein–protein interaction (PPI) network analyses

PPI networks were constructed using the STRING database (version 11.5)^31^ in Cytoscape^30^. Full STRING networks were constructed using a multiple protein query with the default confidence score of 0.4 (medium confidence). STRING network analysis was performed using the STRING web interface. Hub nodes were identified in Cytoscape using the CytoHubba plug-in^32^. The maximal clique centrality (MCC) topological analysis method was used to calculate hub nodes. The top 20 hub nodes were visualised in a sub-network.

### Functional enrichment using g: profiler

Functional enrichment analysis was performed based on a previously published protocol^33^. First, functional enrichment was performed using g: Profiler^34^ (version e109_eg56_p17). The significance threshold was set at 0.05 and the following data sources were used Gene Ontology (GO) molecular function, GO cellular component, GO biological process, KEGG^35^, Reactome, and WikiPathways. If ambiguous query genes were found, these were manually disambiguated by selecting the most appropriate term when prompted and the query was re-run. Results were filtered in terms of the size of the functional category to facilitate the interpretability of the results. The minimum and maximum term sizes were set at 5 and 1000, respectively, to remove excessively small or large gene sets of limited interpretive value. All results were downloaded for further processing. For functional enrichment analysis, a custom background set based on the following criteria was used. Transcripts that are expressed in skeletal muscle and detected in this RNA-sequencing experiment were identified using the normalised read counts in non-PAD controls and CLTI samples. The threshold cut-off was set as minimum median normalised read count of any DEG. Any transcript that had a normalised read count greater or equal to this value was included i.e. only transcripts that were detected at a level that would have a chance at being identified as a DEG were included as has been previously suggested^36^. “Custom over annotated genes” was selected, such that only annotated genes that were also in the background list were used as background genes.

### Enrichment mapping using cytoscape

Visualisation of functional enrichment data from the g: Profiler output was performed using Cytoscape v3.10^30^ as well as the EnrichmentMap v3.3.6^37^, AutoAnnotate v1.4.1^38^, and ClusterMaker2 v2.3.4^39^ plug-ins. First, using EnrichmentMapping, a new enrichment map was created. The analysis type was set as “Generic/g: Profiler”. The enrichments file from the g: Profiler output and the GMT file downloaded from g: Profiler were loaded into the tool. The FDR cut-off was set to 0.05. Other parameters were left at default settings. To identify major “biological themes”, the resulting enrichment map network was then clustered using AutoAnnotate, which in turn uses ClusterMaker2 and the Markov Cluster (MCL) algorithm^40^. Using the default settings in the “Quick Start” tab, the network was annotated. The automatically generated cluster names were edited manually to reflect the biological theme of the cluster. The clusters were manually arranged to separate sub-networks. For visualisation, clusters with two terms or fewer were manually removed unless connected to a larger cluster.

### Animals

 15-week-old male BALB/c nude mice were purchased from Janvier Labs, (France) and were housed in the Bio-Resource Unit (BRU) at the University of Galway, with monitoring and support provided by qualified animal technicians and a veterinary surgeon. All animal experiments were carried out in compliance with the Directive 2010/63/EU and in accordance with ARRIVE guidelines. Ethical approval was granted by the Animal Care Research Ethics Committee (ACREC) at the University of Galway (Ireland) and appropriate individual and project authorizations were granted by the Health Products Regulatory Authority in Ireland (AE19125 /P076). At day 7 post-HLI, mice were euthanised by overdose of anaesthesia (225 mg/kg ketamine and 1.5 mg/kg medetomidine solution injected subcutaneously) followed by cervical dislocation, and their body weights were recorded.

### Induction of HLI and assessment of limb function

 Unilateral hindlimb ischaemia (HLI) was induced in BALB/c nude mice by ligation of the femoral artery distal to the deep femoral artery, as previously described by our group^41^. Animals were anaesthetised with 75 mg/kg ketamine and 0.5 mg/kg medetomidine (Domitor 10) solution injected subcutaneously. Anaesthesia was partially reversed with atipamezole (5 mg/kg). The mice received analgesia (0.05–0.1 mg/kg of buprenorphine 8–12 h for three days, and as required thereafter) and prophylactic antibiotic (0.1 mg/kg of Enrofloxacin/Baytril) was also given once post-operatively. The laser Doppler Imager (LDI) (MoorLDI V6.0, Moor Instruments, Axminster, UK) was used to confirm drop in blood flow perfusion after HLI surgery as previously described^41^. Blood flow was measured in the soles of both feet, before (Pre-) and immediately after HLI surgery (Post-), and 7 days post-surgery, at which point, mice were humanely euthanised. Additionally, control mice of similar age not undergoing HLI surgery were euthanised. Assessment of limb functionality (ambulatory score) was performed on days 3 and days 7 post-surgery. The ambulatory score ranged from 0 to 3 depending on the limb mobility (3 = dragging the foot; 2 = no dragging the foot but no plantar flexion; 1 = plantar flexion but no flexion of toes; 0 = flexion of toes to resist traction on the tail similar to the non-operated foot)^42^ .

### Tissue dissection and preparation

 The calf muscles from the ischaemic (right) and non-ischaemic (left) hindlimbs of HLI mice and the right hindlimb of non-HLI mice were carefully dissected, and their wet weight was recorded. Calf muscle mass was calculated as a percentage per body weight (calf muscle weight [g]*100/ body weight [g]). Proximally, the soleus and gastrocnemius were separated and the proximal ~ 1/3 of both muscles were removed, snap-frozen in LN2 and stored at -80 °C separately for molecular analysis. Distally, the soleus and gastrocnemius were kept intact and were fixed in 10% formalin for 48 h, processed using the Epredia™ Excelsior™ AS Tissue Processor and embedded in paraffin blocks.

### Histopathological assessment of ischaemia severity

 Tissue cross-sections of 5 μm thickness were taken from the mid-belly of the dissected calf muscles and rehydrated through a series of ethanol grades before staining with Haematoxylin and Eosin (H&E) and Mallory Trichrome staining using standard protocols. A semiquantitative scoring system was used to assess histopathological parameters of muscle ischaemia, including inflammation, fibrosis, necrosis, muscle de-/regeneration and adipocyte infiltration, as previously described^41^. Each parameter was independently scored using an analogue scale ranging from 0 to 3. A cumulative ischaemic severity score (cISS) was also obtained from the sum of each individual score.

### Immunohistochemistry

 Myofibre outline and connective tissue were stained with 5 µg/ml of fluorescein-conjugated wheat germ agglutinin (WGA) (Vector Laboratories) for 10 min and cell nuclei were counterstained with DAPI (Fluroshield™, Sigma-Aldrich) and scanned at 10x magnification using an EVOS M7000 Slide Scanner Microscope (Invitrogen).

### RNA isolation

 Total RNA was isolated from a portion of the proximal gastrocnemius muscle using the mirVana miRNA Isolation kit, with phenol (Invitrogen AM1560) according to the manufacturer’s instructions. RNA was quantified using a Qubit™ 4 fluorometer (Invitrogen) and the associated Qubit™ RNA broad-range (BR) assay kit (Invitrogen, Q10211).

### miRNA RT-qPCR

 10 ng of total RNA was reverse transcribed using the Qiagen miRCURY LNA RT kit according to the manufacturer’s protocol. 1µL of each RNA sample was diluted to 5 ng/µL by adding an appropriate volume of RNase-free H_2_O. cDNA was diluted 1:60 and subsequently used in qPCR reaction using the miRCURY LNA qPCR SYBR Green kit and primer assays (See Major Resources Table S1 in supplementary information) on an Applied Biosystems™ StepOnePlus™ Real-Time PCR System. Relative expression of target miRNAs was normalised to miR-27b expression using the 2^−ΔCт^ method.

### mRNA RT-qPCR

 1000ng of total RNA was used for reverse transcription using the Invitrogen Superscript IV Kit according to the manufacturer’s instructions. qPCR was performed in 10uL reactions using Fast SYBR™ Green Master Mix (Applied Biosystems). 5ng of cDNA was used per reaction, a primer concentration of 200–500 nM and an annealing temperature of 60–62 °C were used. Primer sequences (see Major Resources Table) were retrieved from PrimerBank which have been validated to specifically amplify the target of interest^43^. Samples were run in technical duplicates and a no template control (NTC) was run for each primer pair using molecular grade H_2_O instead of a cDNA sample. Relative expression of target mRNAs was normalised to the geometric mean of *Rpl13a* and *Gapdh* expression using the 2^−ΔCт^ method.

### Statistical analysis

All statistical analyses were performed using GraphPad Prism Version 9.4.1. In general, data were presented as mean ± standard deviation (SD) for continuous variables or median ± interquartile range (IQR) for ordinal variables. D’Agostino & Pearson test was used to determine whether sample data came from a normally distributed population. Mean differences between the three groups were tested statistically with a one-way ANOVA followed by Tukey’s multiple comparison test. Statistical significance was assigned at p-value ≤ 0.05.

## Results

### miRNA-target interaction enrichment analysis

**Fig. 1:**
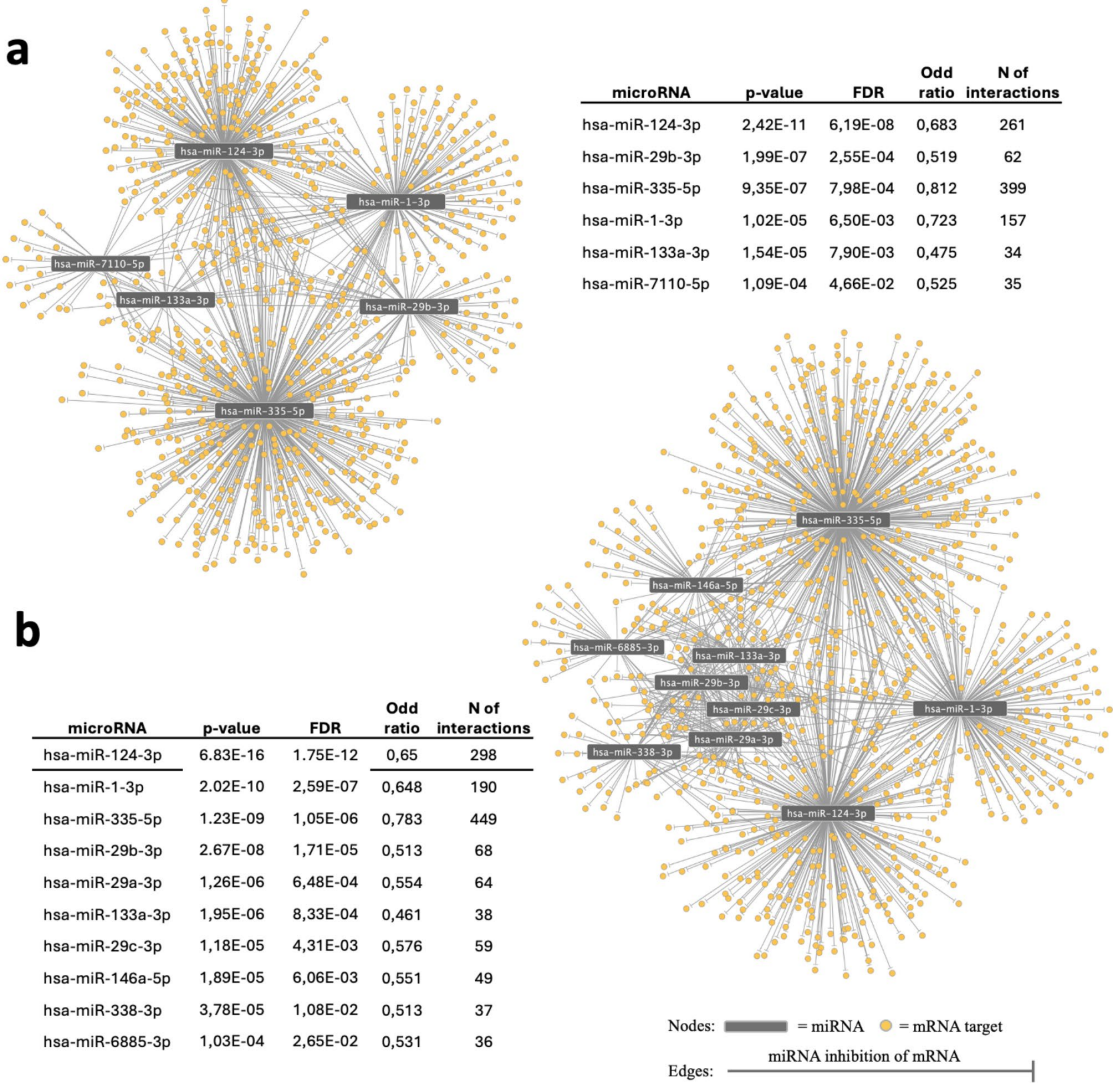
Significantly overrepresented miRNAs amongst UpDEGs in CLTI, IC and non-PAD muscle. A network representation of the enriched MTIs in upregulated DEGs in skeletal muscle from patients with CLTI and IC. The MIENTURNET tool was used to create an MTI network of the significantly enriched MTIs in CLTI vs. non-PAD muscle (**a**) and CLTI vs. IC muscle (**b**) using the miRTarBase database for experimentally validated MTIs. No MTI network was created for IC vs. non-PAD muscle as there were no significantly enriched MTIs when comparing these two groups. The MTI network was visualised using Cytoscape. miRNAs are represented by grey nodes and mRNA targets are represented by yellow nodes. Edges represent validated MTIs. For more information on miRNA targets please refer to Supplementary Information S1.

The muscle transcriptomic dataset obtained from Ryan et al. identified a total of 3,627 genes expressed differentially in CLTI vs. non-PAD control; 3,999 genes differentially expressed in CLTI vs. IC; and 397 DEGs in IC vs. non-PAD controls^12^. Of these, 2261 were upregulated (UpDEG) and 1,366 were downregulated (DownDEG) in CLTI vs. non-PAD controls; 2,514 were upregulated and 1,485 were downregulated in CLTI vs. IC; 182 were upregulated and 215 were downregulated in IC vs. non-PAD. MTI enrichment analysis was performed using MIENTURNET based on the validated MTIs in the miRTarBase database. MTI enrichment of UpDEGs identified 6 over-represented miRNAs in CLTI vs. non-PAD controls and 10 over-represented miRNAs in CLTI vs. IC (Fig. 1). No miRNAs were found to be significantly enriched when comparing the DownDEGs of CLTI vs. non-PAD or CLTI vs. IC, or when comparing the UpDEGs or DownDEGs of IC vs. non-PAD controls (See Supplementary Material S1). An MTI network of the significantly enriched miRNAs and the corresponding upregulated targets was constructed showing a total of 948 MTIs for the CLTI vs. non-PAD group and 1,288 MTIs for the CLTI vs. IC group (Fig. 1). A list of transcripts targeted by each miRNA can be accessed in Supplementary Material S1.

### Severe calf muscle pathology in HLI

**Fig. 2:**
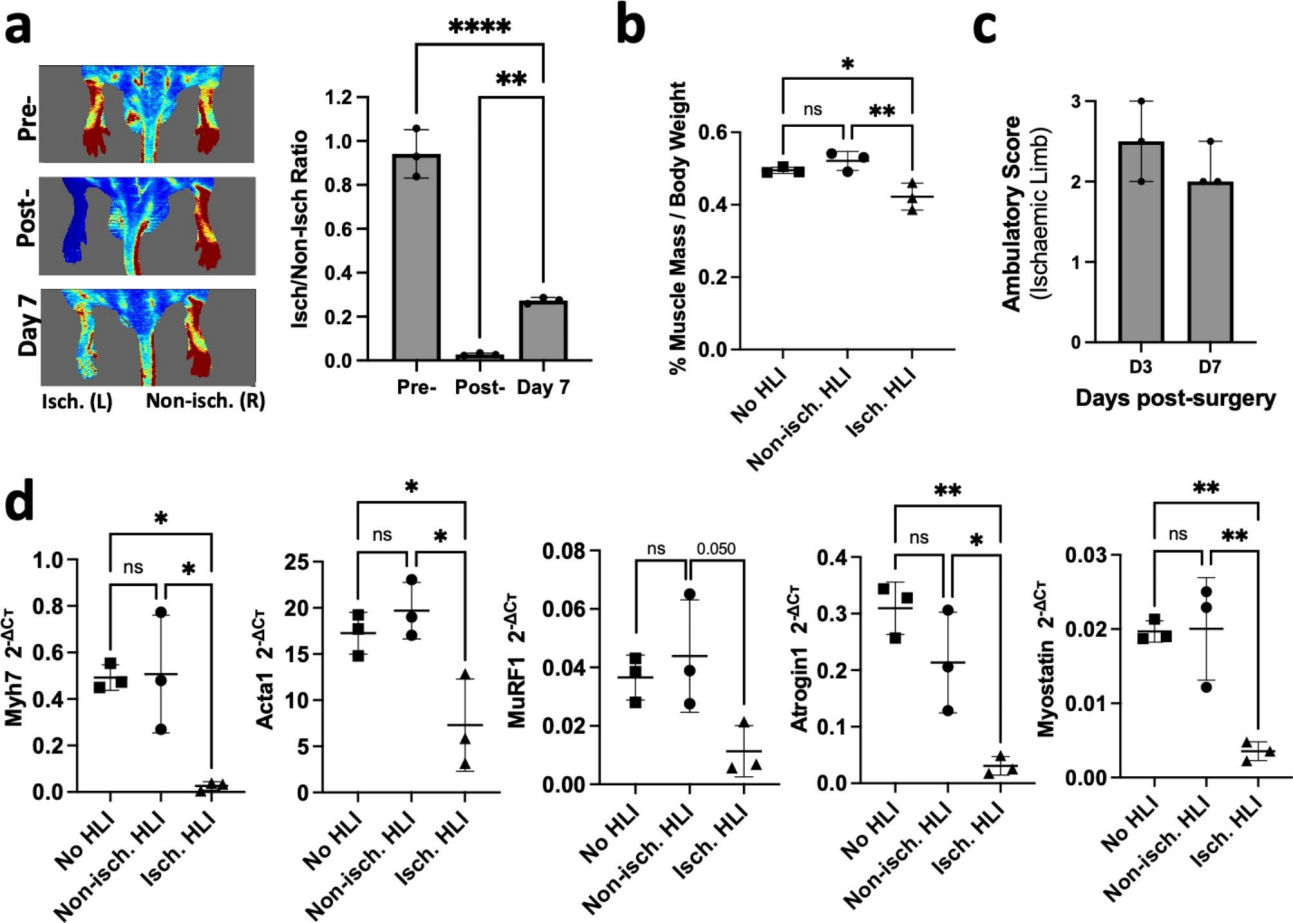
Severe calf muscle pathology 7 days post-HLI. (**a**) Blood flow perfusion of mice foot using Laser Doppler Imaging. Colour-coded images displayed poor perfusion as dark blue, and the highest perfusion level was displayed as red. Data are mean ± SD. **p < 0.01, *p < 0.05 (one-way ANOVA, Tukey’s multiple comparisons test). (**b**) Percentage of calf muscle wet weight per body weight. Data are mean ± SD. **p < 0.01, *p < 0.05 (one-way ANOVA, Tukey’s multiple comparisons test). (**c**) Assessment of limb functionality using the ambulatory score (3 = dragging the foot; 2 = no dragging the foot but no plantar flexion; 1 = plantar flexion but no flexion of toes; 0 = flexion of toes to resist traction on the tail similar to the non-operated foot). (**d**) Expression levels of Myh7, Acta1, MuRF1, Atrogin1 and Myostatin assessed by RT-qPCR. Cт values were normalised to the geometric mean of Rpl13a and Gapdh using the 2^−ΔCт^ method. Data are presented as mean ± SD. *p < 0.05, **p < 0.01 (one-way ANOVA, Tukey’s multiple comparison test).

A preclinical HLI model was used to validate the bioinformatically identified miRNAs in skeletal muscle ischaemia, as done in previous studies^44^. HLI surgery immediately reduced the blood flow perfusion to 5% in the ischaemic limb. Blood flow recovery increased over time, but it was still impaired on day 7 (Fig. 2a). At 7 days post-HLI, a significant muscle atrophy and functional decline were observed, as indicated by an overall decrease in the percentage of calf muscle weight per body weight in ischaemic hindlimbs vs. the contralateral limb and no-HLI control mice (Fig. 2b), and a poor ambulatory score (Fig. 2c). We then analysed the expression levels of myosin heavy chain 7 (Myh7) and alpha skeletal muscle actin (Acta1), which are two genes related to skeletal muscle phenotype and structure, and therefore related to muscle mass^45,46^ (Fig. 2d). Results showed reduced expression of Myh7 and Acta1 in the ischaemic limb vs. the contralateral non-ischaemic limbs and no-HLI control mice, an indicator of muscle mass loss. We also analysed the expression of gene regulators of muscle growth, including the muscle-specific ubiquitin ligases muscle RING-finger protein-1 (MuRF1) *(*also known as Trim63)^47^, Atrogin1 (also known as Fbxo32)^48^ and myostatin (Mstn)^49^. The expression of these three genes were significantly suppressed in the ischaemic limb compared to the contralateral non-ischaemic limb (MuRF1, *p* = 0.050) (Fig. 2d). This gene expression data suggest that the normal atrophic and myogenic transcriptional programs are dysregulated 7 days after ischaemic injury.

**Fig. 3:**
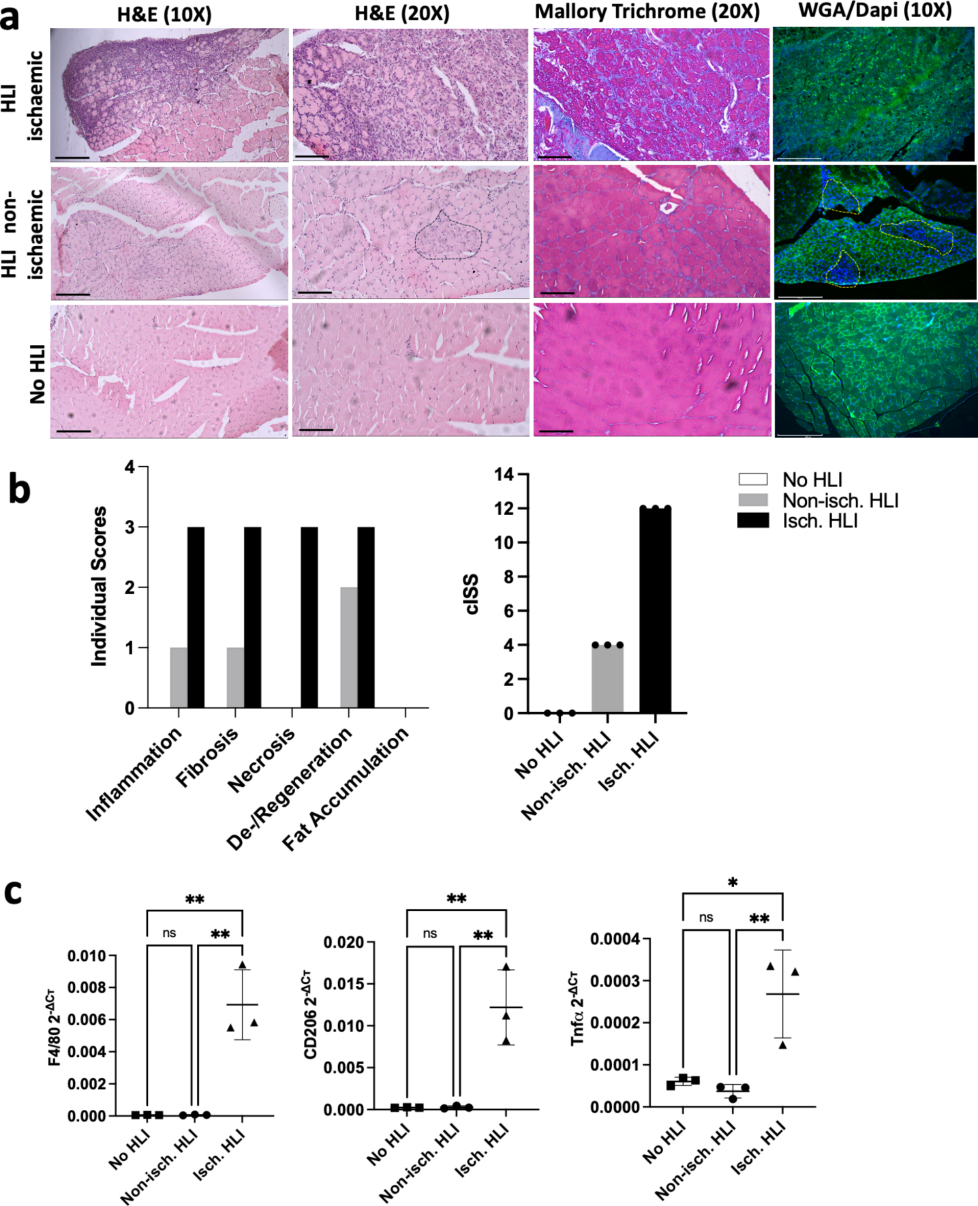
Assessment of ischaemia-induced skeletal muscle damage. (**a**) Representative images of skeletal muscle sections stained with H&E, Mallory’s Trichrome stain and muscle fibre morphology by WGA/DAPI staining. Scale bar 10X = 250 μm; 20X = 50 μm (Brightfield images), 10X = 275 μm (Fluorescent image), WGA (green), DAPI (blue). (**b**) Semiquantitative histopathological assessment of ischaemic damage: bi) individual scores for each histopathological parameter assessed; bii) cumulative ischaemia severity score (cISS). Data are median ± IQR. (**c**) Expression levels of F4/80, CD206 and TNFα assessed by RT-qPCR. Cт values were normalised to the geometric mean of Rpl13a and Gapdh using the 2^−ΔCт^ method. Data are mean ± SD. *p < 0.05, **p < 0.01 (one-way ANOVA, Tukey’s multiple comparison test).

Histologically, the skeletal muscle architecture was dramatically perturbed after ischaemic injury (Fig. 3a). The extent of muscle ischaemia-induced damage was quantified as previously^41^ (Fig. 3b). The ischaemic calf muscle presented with severe inflammation characterised by high levels of Tnf and widespread intermuscular mononuclear cell infiltration across large areas of tissue macrophages (F4/80) and M2 macrophages (mannose receptor C type 1, Mrc1 or CD206) (Fig. 3c). Inflammation was accompanied by a significant loss of muscle fibre integrity architecture and fibre degeneration along with necrotic fibres affecting large regions of the muscle (Fig. 3a, WGA staining), and included a marked presence of intermuscular collagen fibrillar material deposition (Fig. 3a, blue in Mallory Trichrome staining). Interestingly, we observed mild inflammation and fibrosis focalised to several myofibers in a small region of the muscle in the contralateral non-ischaemic limbs (Fig. 3a). The myofibres affected showed signs of undergoing regeneration as shown by their small size and centralised nuclei (dotted area in Fig. 3a). Abnormal intermuscular adipocyte infiltration was absent at this timepoint in all the muscles analysed. A final total cumulative ischaemia severity score (cISS) was calculated for each animal by adding up the individual scores (Fig. 3b). Overall, ischaemic muscles had a cISS = 12 (i.e. severe ischaemic damage) while non-ischaemic contralateral limb had a cISS = 4 (mild ischaemic damage).

### miRNA dysregulation in skeletal muscle ischaemia

**Fig. 4:**
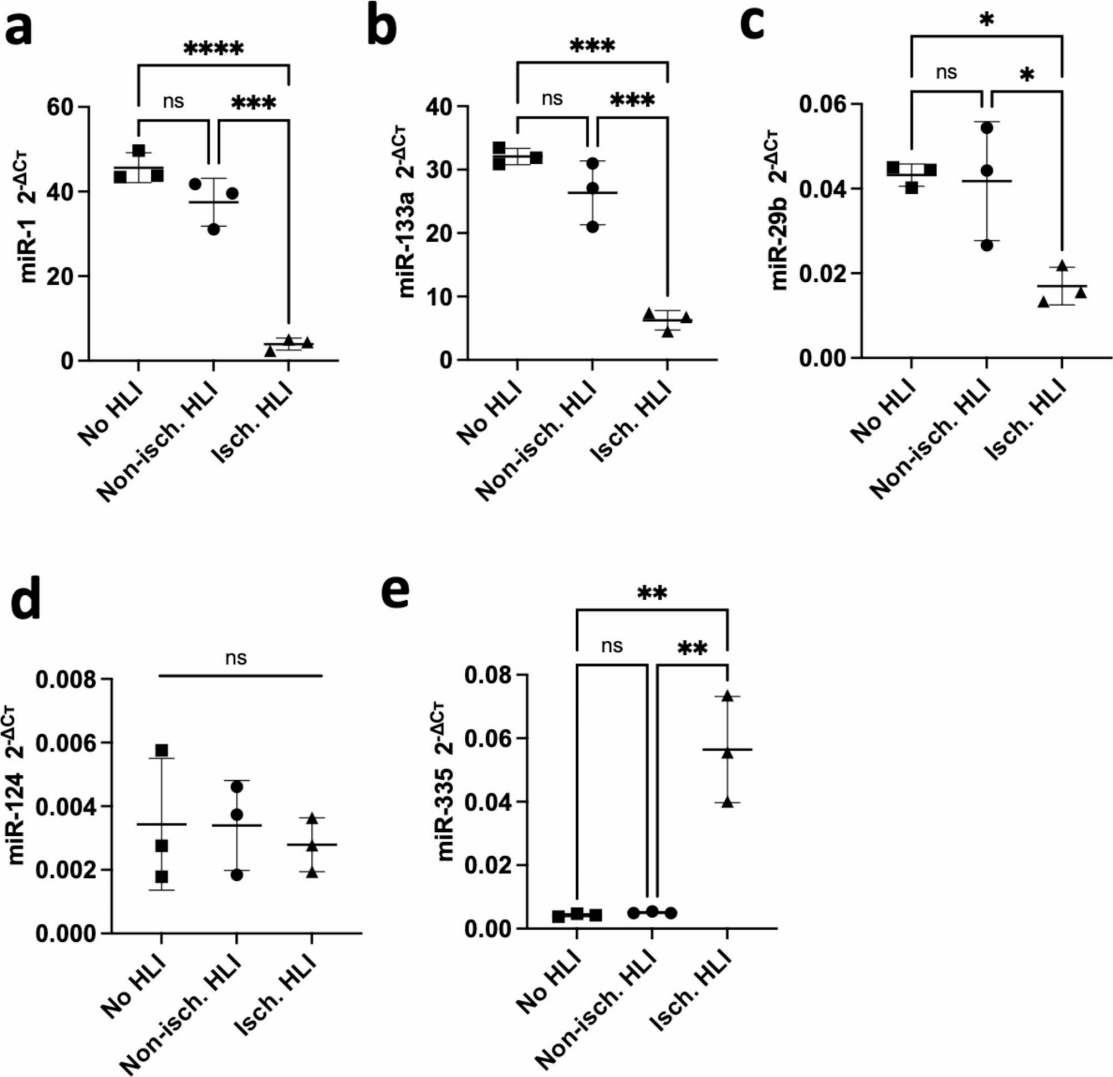
Validation of over-represented miRNAs in CLTI skeletal muscle using HLI mouse model. The expression levels of (**a**) miR-1-3p, (**b**) miR-133a-3p, (**c**) miR-29b-3p, (**d**) miR-124-3p and (**e**) miR-335-5p were assessed in the gastrocnemius muscle of no HLI (control) mice and of HLI mice *via* RT-qPCR. miRNA Cт values were normalised to miR-27b using the 2^−ΔCт^ method. Data are presented as mean $\:\pm\:$SD. * *p* < 0.05, ** *p* < 0.01, *** *p* < 0.001. One-way ANOVA and Tukey’s multiple comparison test.

Since MTIs of identified miRNAs were over-represented among UpDEGs in ischaemic muscle, it was hypothesised that the levels of these miRNAs would be decreased. RT-qPCR was performed on RNA isolated from the gastrocnemius muscle of the ischaemic and non-ischaemic limbs of mice that underwent the HLI procedure, and control mice that did not undergo the HLI. The results showed a significant and marked ischaemia-induced decrease in miR-1, miR-133a, and miR-29b in the ischaemic limbs compared to the contralateral non-ischaemic limbs (*p* = 0.0001, *p* = 0.0005, *p* = 0.0292, respectively) and the no-HLI control mice (*p* < 0.0001, *p* = 0.0001, *p* = 0.0230, respectively) (Fig. 4a,b,c). Contrary to the hypothesis, miR-124 was not significantly dysregulated in the ischaemic limbs compared to the contralateral non-ischaemic limbs (*p* = 0.8792) or the control no-HLI control mice (*p* = 0.8662) (Fig. 4d). miR-335 expression was significantly higher in the ischaemic limbs than the contralateral non-ischaemic limbs (*p* = 0.0015) and the control no-HLI control mice (*p* = 0.0014) (Fig. 4e). miR-7110 was not assessed as it was not annotated in mice in the miRBase database^50^.

### ***miR-1***,*** miR-133a***,*** and miR-29b functional enrichment analysis***

**Fig. 5:**
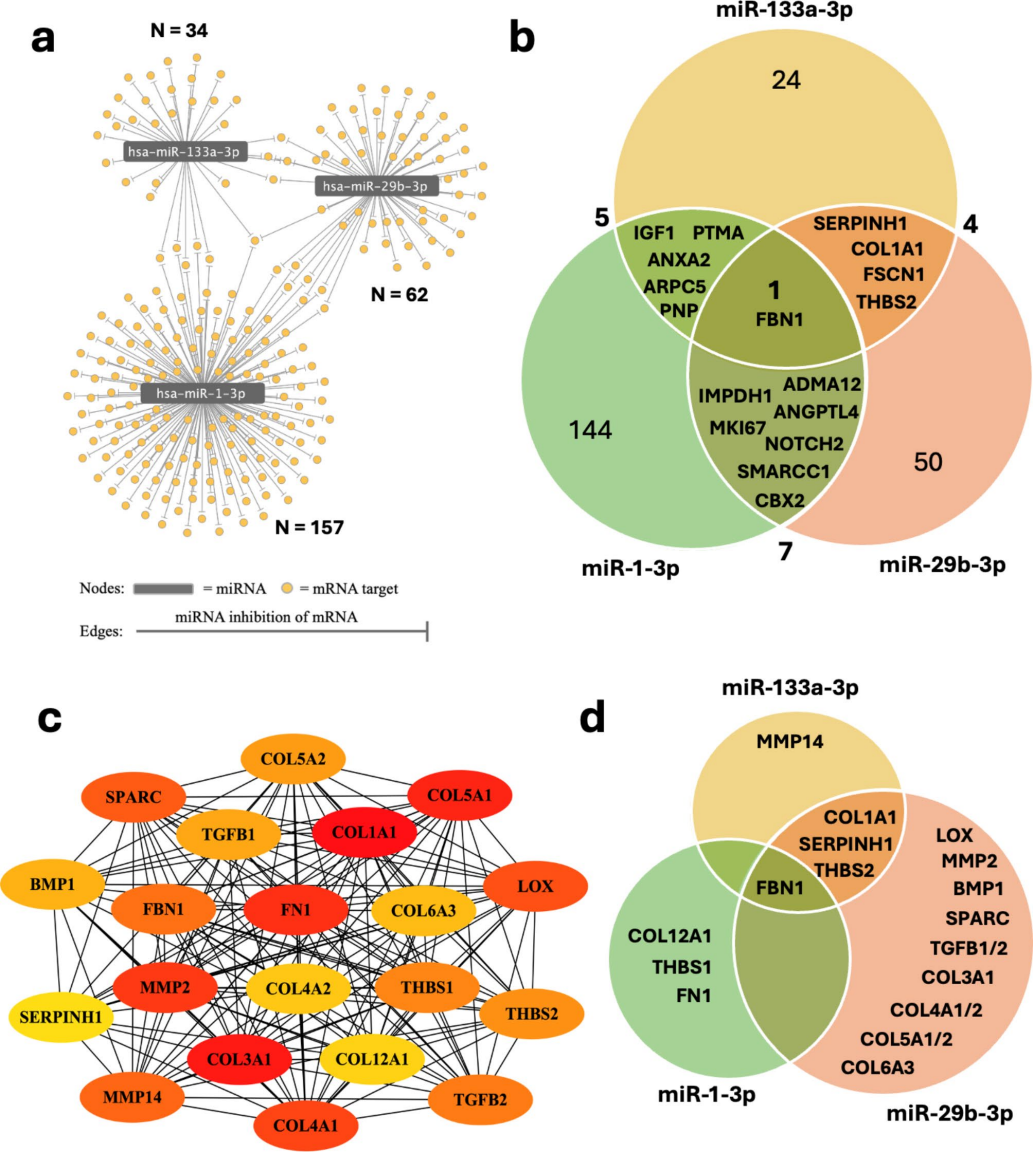
Subnetwork analysis of miR-1, miR-133a and miR-29b targets. (**a**) Subnetwork of the MTI network in Fig. 1 of miR-1, miR-133a, and miR-29b and their targets which are also upregulated in CLTI was created using Cytoscape. miRNAs are represented by grey nodes and mRNA targets are represented by yellow nodes; edges represent validated MTI from the miRTarBase database. N = total miRNA targets. (**b**) Venn diagram representation of miR-1, miR-133a and miR-29b shared targets. (**c**) The hub nodes of the PPI network in Fig S1 were identified in Cytoscape using CytoHubba and the MCC topological analysis method. The top 20 hub nodes were used to create a subnetwork. Node colour indicates hub node essentiality, red indicates greater hub node rank and yellow indicates lesser hub node rank. (**d**) Venn diagram representation of pro-fibrotic transcripts selected from the top 20 hub nodes which are targeted by miR-1, miR-133a and/or miR-29b.

**Fig. 6:**
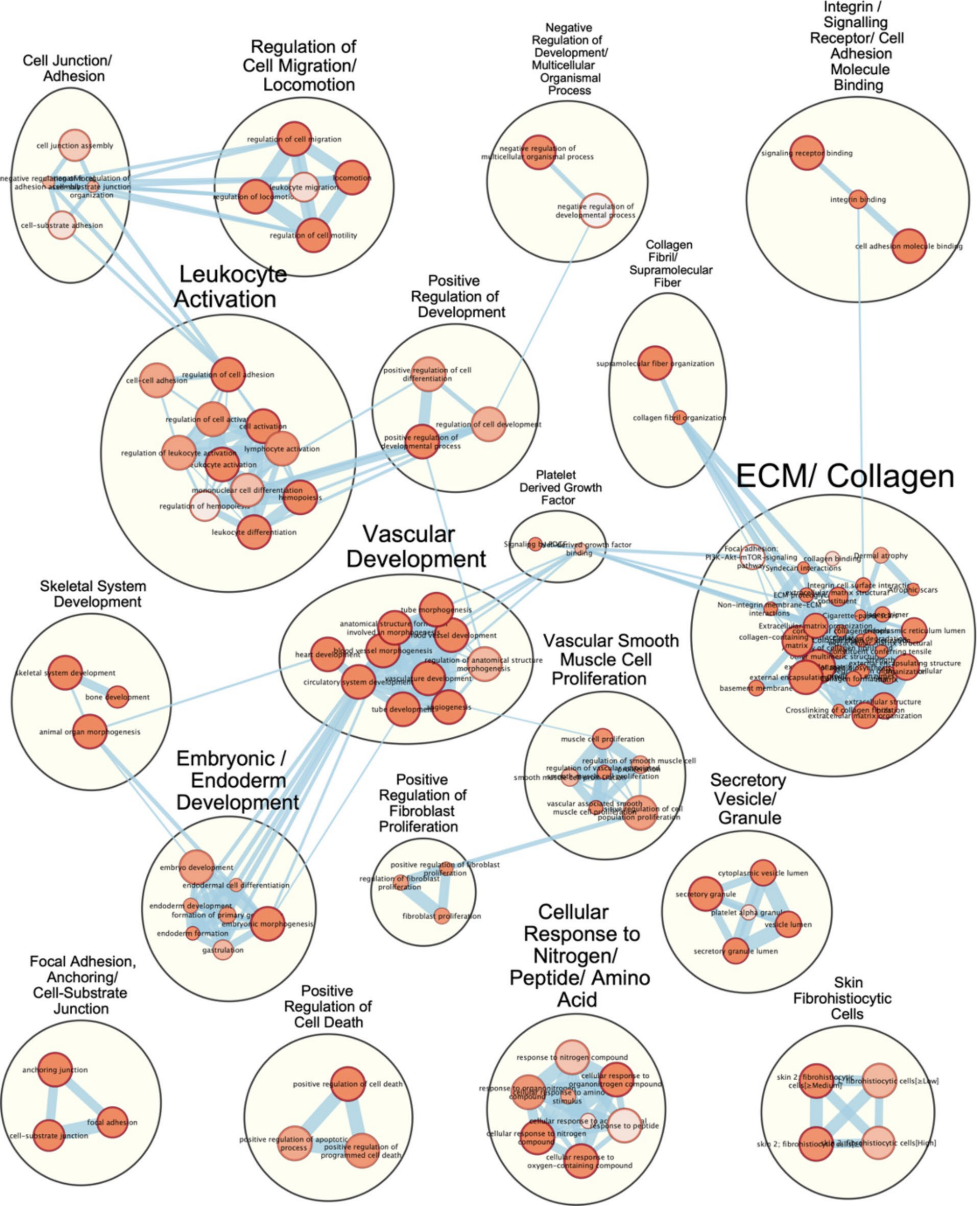
Enrichment map of miR-1, miR-133a, and miR-29b targets upregulated in CLTI. Functional enrichment analysis was performed on the targets of miR-1, miR-133a, and miR-29b that are upregulated in CLTI gastrocnemius using g: Profiler. EnrichmentMapping was performed using the EnrichmentMap plugin in Cytoscape. Cluster labels created using the AutoAnnotate plugin were manually edited. Nodes represent enriched pathways identified using g: Profiler. Edges represent, and are weighted by, pathway gene set overlap. Node colour is mapped to pathway enrichment significance (orange = lower Q value and white = higher Q value). Node size is mapped to gene set size.

As hypothesized, miR-1, miR-133a, and miR-29b expression was downregulated in ischaemic mouse muscle. It was then considered that these three miRNAs may co-operatively regulate the skeletal muscle pathology in CLTI. The mRNAs that were: (a) targeted by one or more of these miRNAs, and (b) upregulated in CLTI patient skeletal muscle, were further investigated. First, an MTI subnetwork was created for the three differentially expressed miRNAs and the network characteristics were assessed (See Supplementary Information S1) (Fig. 5a). Notably, most mRNAs were targeted by one miRNA and a minority were targeted by two miRNAs with only one targeted by all three miRNAs. A list of shared targets are represented in (Fig. 5b) and the full list of miRNA targets are available as Supplementary Information S1. To understand the functional interactions of these enriched targets, PPI network analyses were performed using STRING. The STRING PPI network consisted of 235 nodes with 965 edges, and the expected number of edges was 362 with a corresponding PPI enrichment p-value of < 1*10^−16^ indicating a significant degree of interaction between the input targets (Fig. S1). The most important of these interacting nodes, i.e. the “hub nodes”, were then identified by the Mean Clique Centrality algorithm using CytoHubba. The top 20 hub nodes are displayed in Fig. 5c and they included many extracellular matrix (ECM) components such as collagens I, III, IV V, and VI as well as fibronectin-1 (Fbn1) and fibrin-1 (Fn1), among others. Most of the targets within this sub-network were targeted by miR-29b alone. Fbn1 was targeted by the three miRNAs while Col1a1, Serpinh1 and Thbs2 were targets of both miR-29b and miR-133a, and Col12a1, Thbs1 and Fn1 were targeted by miR-1 alone (Fig. 5d). Functional enrichment analysis was performed on this subset of DEGs and EnrichmentMapping was then obtained (Fig. 6). The largest cluster consisted of terms associated with ECM and collagen which was connected with neighbouring clusters associated with collagen fibrils, integrin/signalling receptor/cell adhesion molecule binding, and platelet-derived growth factor (PDGF). The second-largest cluster was associated with leukocyte activation which connected to positive regulation of development and cell junction/adhesion clusters. The third largest cluster contained terms related to cardiovascular/blood vessel development which had five first-degree neighbouring clusters. These were related to embryonic development, skeletal system/bone development, PDGF, positive regulation of development, and vascular smooth muscle cell (VSMC) proliferation. There was a cluster of terms related to fibroblast proliferation connected to the VSMC proliferation cluster. Other clusters included those related to negative regulation of development, regulation of cell migration, secretory vesicle/granule, focal adhesion, anchoring/cell-substrate junction, positive regulation of cell death, and cellular response to nitrogen/peptide/amino acid. EnrichmentMapping of the targets of individual miRNAs can be accessed in Fig. S2.

### mRNA target validation in the HLI mouse model

**Fig. 7:**
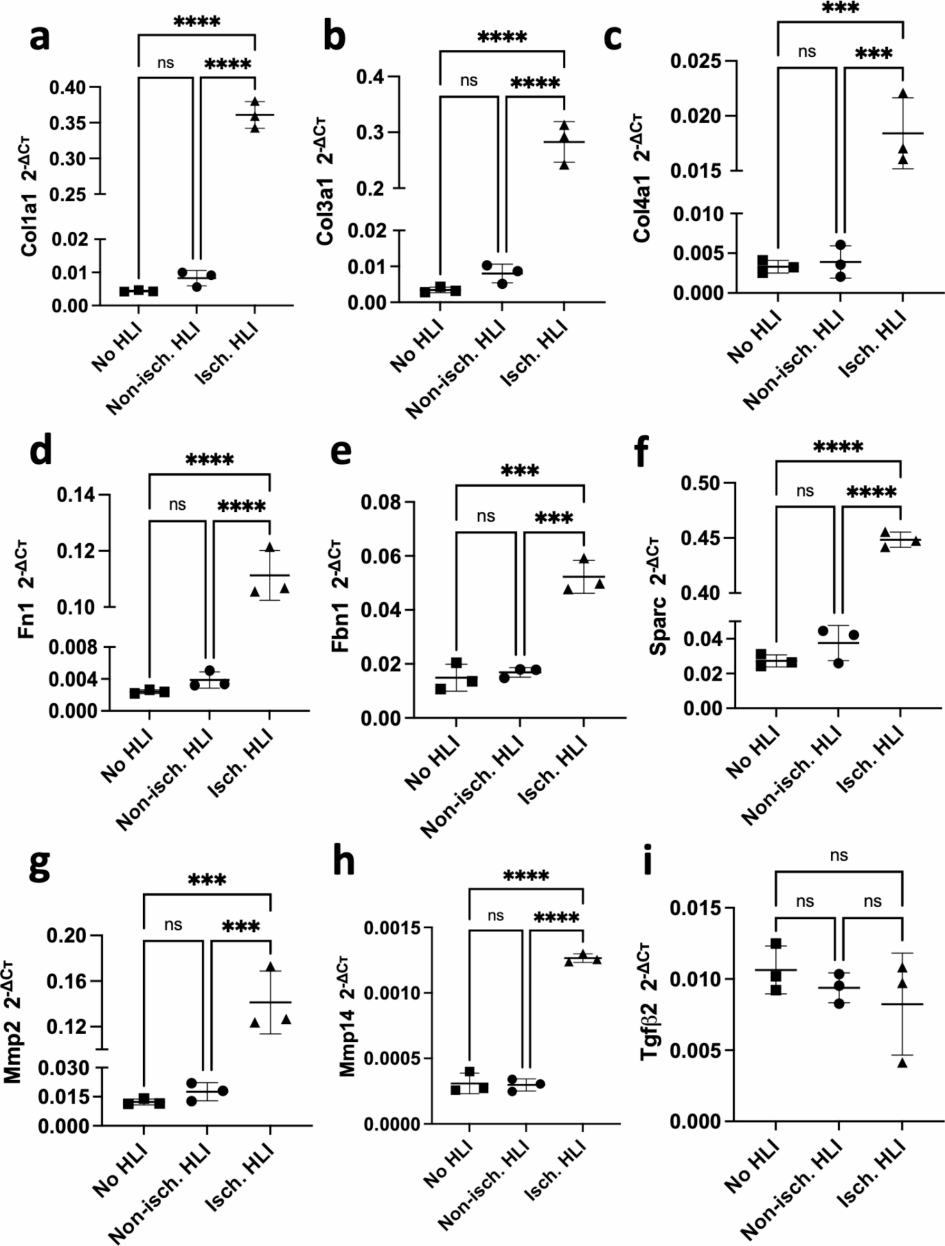
Expression of hub nodes of the miR-1, miR-133a, and miR-29b target PPI network in skeletal muscle ischaemia. The expression levels of (**a**) Col1a1, (**b**) Col3a1, (**c**) Col4a1, (**d**) Fn1, (**e**) Fbn1, (**f**) Sparc, (**g**) Mmp2, (**h**) Mmp14, and (**i**) Tgfβ2 were assessed in the gastrocnemius muscle of no-HLI (control) mice and of HLI mice *via* RT-qPCR. miRNA Cт values were normalised the geometric mean of Rpl13a and Gapdh expression using the 2^−ΔCт^ method. Data are presented as mean $\:\pm\:\:$SD. * *p* < 0.05, ** *p* < 0.01, *** *p* < 0.001. One-way ANOVA and Tukey’s multiple comparison test.

We then validated specific targets of these miRNAs in the HLI mouse model. A sample of transcripts that were targeted by one, two and/or three miRNAs were selected from the top hub nodes from Fig. 5c. All the targets assessed were significantly increased in the ischaemic limbs compared the contralateral non-ischaemic limbs and the no-HLI control mice, except for Tgfb2, which was not changed (Fig. 7). None of the targets analysed were differentially expressed between the non-ischaemic limbs of HLI mice and no-HLI control mice, although there were trends towards increased Col1a1, Col3a1 and Fn1 in the contralateral non-ischaemic vs. no-HLI muscle.

## Discussion

PAD is characterised by a complex, multifactorial skeletal muscle pathology that has devastating effects on patient QOL, with significant morbidity and mortality^2–5^. Despite some progress made in terms of medical and surgical interventions for PAD, the prognosis of CLTI remains poor with unacceptably high rates of amputation^51^. Furthermore, novel investigational approaches (e.g. gene therapy with proangiogenic agents and stem cell-based therapies) have not demonstrated significant benefits in promoting CLTI limb salvage^52,53^. One likely reason for this is the complex pathology of CLTI which is not affected by the dysfunction of a single gene; rather, it is characterised by broad gene dysregulation resulting in derangement of signalling pathways, processes, and networks in skeletal muscle^12,15,16^. Therefore, understanding the molecular regulation of skeletal muscle pathology in CLTI is imperative and will help in the rational design of novel therapeutics with a greater likelihood of success than previously attempted therapies.

Considering the broad transcriptomic dysregulation in CLTI muscle^12^, we investigated whether miRNAs could regulate these DEGs. We performed MTI enrichment analysis on the DEGs using MIENTURNET to identify miRNAs which may regulate significant portions of the dysregulated transcriptome in CLTI muscle. The underlying rationale for this was two-fold. First, it may further elucidate the underlying molecular pathology of CLTI-induced skeletal muscle pathology. Second, miRNAs identified in such a manner may represent novel therapeutics that could be delivered (or inhibited) as novel therapeutics to influence pathological gene expression and restore homeostasis in the ischaemic limb. For the first time, our bioinformatic analysis has identified a CLTI-associated miRNA dysregulation signature. Among the investigated UpDEGs, we identified six over-represented miRNAs in CLTI vs. non-PAD controls (miR-124-3p, miR-1-3p, miR-133a-3p, miR-29b-3p, miR-335-5p, and miR-7110-5p). When comparing CLTI vs. IC, we obtained a list of 10 over-represented miRNAs: miR-124-3p, miR-1-3p, miR-133a-3p, miR-29b-3p and miR-335-5p, which were also obtained when comparing CLTI vs. non-PAD controls, as well as others including miR-29a-3p, miR-29c-3p, miR-338-5p, miR-146a-5p, and miR-6885-3p. No over-represented miRNAs were found when comparing the IC and non-PAD controls (See Supplementary Information S1).

We then used the preclinical mouse model of CLTI to validate the predicted downregulation of miRNAs identified in CLTI vs. non-PAD controls. The rationale for choosing this group was that, in vivo, we compared skeletal muscles severely affected by ischaemic injury with control non-ischaemic muscles. First, skeletal muscle pathology was confirmed in the HLI model 7 days post-ischaemia induction. The presence of significant skeletal muscle atrophy was demonstrated by a reduction in calf muscle mass and impaired limb function, as well as downregulation of muscle mass-related genes (Myh7 and Acta1*)*. Furthermore, Atrogin1, MuRF1 and Mstn, known regulators of muscle mass^47^, were also decreased in the ischaemic limb (Fig. 2c). A similar finding has been previously reported where these markers were significantly suppressed in BALB/c mice at 7 and 28 days post-HLI^42,54^. Histologically, all ischaemic muscle analysed presented with severe inflammation, necrosis, and fibrosis with significant loss of muscle fibre integrity, which is consistent with human data^2,55,56^. We observed focalised mild inflammation and fibrosis in the contralateral non-ischaemic limb of HLI mice which was accompanied by myofibre regeneration. This phenotype seems to return to normal appearance at 28 days post-HLI surgery^42^. It is possible that this transient inflammation and fibrosis observed at 7 days post-HLI but not at day 28-post HLI may be related to a transient muscle damage induced by an extra work effort^57,58^ taken by the non-ischaemic leg to compensate for the ambulatory impairment in the ischaemic limb (Fig. 2b), which may naturally resolve as the ischaemic limb becomes more functional and able to carry the mouse weight. This is an interesting observation that may be important to consider when using this preclinical animal model.

We then investigated whether the specific miRNAs were downregulated as predicted by our bioinformatics analysis. The most significantly enriched miRNAs identified by our bioinformatics analysis was miR-124-3p (Fig. 1). Functional enrichment analysis of DEGs targeted by miR-124-3p identified enrichment for terms associated with ECM structure and collagen as well as vascular development and angiogenesis (Fig S3a). This is supported by previously published literature, where miR-124-3p has been shown to regulate angiogenesis in an HLI model^59^. Here, delivery of miR-124 mimics in an HLI model inhibited perfusion recovery and decreased capillary density at day 14, while antagomiRs had the opposite effect in both of these measures^59^. RT-qPCR validation of the HLI model on day 7 post-HLI revealed that miR-124 was not dysregulated. Interestingly, Shi et al. reported that miR-124 is transiently increased immediately in response to HLI, with a peak in expression at day 2 and restoration to pre-ischaemic levels by day 3^59^, which supports our findings.

The identification of miR-29b is interesting as it is a well-known fibrosis-associated miRNA, together with miR-29a and miR-29c. The miR-29 family has been shown to be involved in inhibiting ECM synthesis indicating its antifibrotic function^60^. Despite the relatively low number of input genes (Fig. 1), there was a marked degree of functional enrichment among the targets related to the underlying fibrotic mechanisms (Fig S2e). The data presented here contributes to the already known role of miR-29b as a “fibromiR”. We hypothesized that in the ischaemic limb, miR-29b may directly regulate fibrosis-associated pathology, a hallmark feature of CLTI^13^, by targeting ECM-associated transcripts. We validated the downregulation of miR-29b in skeletal muscle and upregulation of its ECM-related target mRNAs (Col1a1, -3a1, -4a1, Fbn1, Sparc and Mmp2, Fig. 7) in response to ischaemic injury, at a time point where intramuscular fibrosis was observed histologically. Other studies have reported decreased levels of miR-29b in fibrosis^61–64^, which support our findings. In addition, the decreased expression in circulation is associated with increased mortality in patients with pulmonary fibrosis^65^. Functional enrichment analysis also identified enrichment for terms associated with arterial dissection/aneurysm and vascular development/angiogenesis and interconnection with fibrosis-associated clusters (Fig S2e). This may be indicative of the fibrotic changes seen in the microvasculature of the PAD/CLTI muscle^66^, in which VSMCs show increased TGFβ levels and collagen deposition. miR-29b has been investigated as a potential anti-fibrotic agent, particularly in the context of pulmonary fibrosis^67^. Delivery of miR-29b mimics has also demonstrated pre-clinical and early clinical efficacy in cutaneous fibrosis (drug name: Remlarsen or MRG-201)^63^. While miR-29b has not been delivered in an HLI model, its family member, miR-29a has been investigated in this context. In murine diabetic HLI models, inhibition of miR-29a enhanced perfusion recovery, muscle regeneration and function, and capillary density by day 21^68,69^. However, while ischaemia induced a decrease in miR-29a expression in the gastrocnemius, diabetic HLI was associated with an increase in miR-29a vs. non-diabetic HLI^68^. This observation was consistent in humans with and without diabetes^68^. The molecular mechanisms by which hyperglycaemia impairs miR-29a expression is not well known. The confounding effects of metabolic dysfunction in the context of ischaemia may have specific effects on miRNA dysregulation that must be considered.

miR-1 and miR-133a are two of the canonical “myomiRs” and were also found to be CLTI-specific (Fig. 1). myomiRs are muscle-enriched miRNAs with key roles in the regulation of muscle function and pathology^70–72^. Early work by Chen et al. identified distinct roles for miR-1 and miR-133a in promoting myogenesis and myoblast proliferation, respectively^73^. Despite increasing acknowledgement of the importance of skeletal muscle pathology in CLTI, myomiRs remain relatively understudied in this context. Greco et al. suggested that a decrease in miR-1 levels (and also miR-29c) in response to ischaemia was not seen in isolated myofibres but only in the total muscle bulk. This suggests that the decrease in expression may be due to the loss or atrophy of myofibres and relative over-representation in the cellular mass in the muscle of non-myofibre cell types^74^. Our analysis showed that both miR-1 and miR-133a also targeted ECM components (Fig. 5d) and upregulation of their pro-fibrotic targets Col1a1, Fbn1, Mmp14, Fn1 and Fbn1 was confirmed (Fig. 7). Fibrosis is a key feature of muscle pathology in CLTI, and myogenic progenitor cell dysfunction is also observed^12,75,76^. The clinical implications of this remain to be fully understood; however, recent in vivo studies using knockout models, have indicated that Pax7^+^ myogenic progenitor cells are necessary for regeneration after HLI injury^77^. In the absence of these cells, fibroadipogenic progenitor cells (FAPs) are activated and contribute to ischaemic muscle pathology by increasing adipogenesis^77^. Dysregulation of the adipogenic and fibrotic differentiation of FAPs due to age, trauma, or disease can lead to abnormal intermuscular fat infiltration and excessive fibrosis, resulting in muscle loss and dysfunction^78^. It is possible that ischaemia-induced impairments in muscle progenitor cell (MPC) function and relative reductions in myomiR levels may regulate fibrosis. For instance, in muscle injury, MPCs traffic miR-206 to fibrogenic cells *via* extracellular vesicles (EVs) and thereby regulate ECM deposition in the muscle^79^.

To the best of our knowledge, miR-1 has not been investigated in terms of its ability to regulate recovery in a pre-clinical HLI model. However, a novel role of miR-1 has been recently elucidated, where the loss of miR-1 results in metabolic inflexibility in skeletal muscle leading to a significant reduction of exercise performance associated with an altered pyruvate metabolism^80^. In contrast, miR-133a has been investigated in a pre-clinical model of diabetic HLI^81^, where the delivery of miR-133a was detrimental (and its inhibition was beneficial) in terms of perfusion recovery, and its expression in the skeletal muscle was upregulated compared to that in non-diabetic mice. Here, in a non-diabetic HLI mouse model, we found a marked downregulation of miR-133a. It is possible that the diabetic milieu may alter miR-133a expression in skeletal muscle ischaemia and impact the effect of modulation of this miRNA, similar to what has been observed for miR-29a^68^. This finding raises important concerns for the field of miRNA therapy in cardiovascular disease as DM is one of the most significant risk factors for PAD and differences in the roles of specific miRNAs in vascular diseases with or without DM are not inconsequential.

miR-335 had the highest number of targets upregulated in the CLTI muscle (Fig. 1). Functional enrichment analysis indicated that this miRNA may regulate the inflammatory response in ischaemia (Fig. S2c). In sepsis-induced myocardial injury, miR-335 exerts a protective effect by regulating the inflammatory response^82^. In the peripheral blood of PAD patients, miR-335 was reported to be downregulated^83^.

miR-7110-5p was not assessed in this pre-clinical model as it has not been annotated in mice^50^. Unlike other miRNAs, this is a relatively novel miRNA. However, recent investigations have suggested that miR-7110-5p is downregulated in diabetes^84^ and hypertrophic cardiomyopathy^85^. Additionally, it has been suggested that miR-7110-3p is implicated in pulmonary arterial hypertension^86^. Primer sequences are commercially available for this miRNA, both − 3p and − 5p strands, so investigation in human CLTI samples is warranted.

In summary, several miRNAs with a potential regulatory role in skeletal muscle pathology in CLTI have been identified by MTI analysis. All the miRNAs analysed here shared 100% homology between mice and humans except miR-7110-5p which is only annotated in humans (Fig S3). Furthermore, the vast majority of miRNA targets, including the ones analysed here are conserved between mice and humans (Fig S3). In pre-clinical validation, three of these miRNAs (miR-1, miR-133a, and miR-29b) were differentially expressed in the HLI model in the opposite direction to their targets in patient samples. Therefore, we hypothesised that these three miRNAs may represent a signature of skeletal muscle pathology in CLTI. To understand the potential role of these three miRNA panels in skeletal muscle ischaemia, we created a sub-MTI network of miR-1, miR-133a, and miR-29b and their targets that were upregulated in the CLTI muscle (Fig. 5). A PPI network was constructed to understand the functional association between the targets of these miRNAs in skeletal muscle ischaemia. Our results revealed a significant degree of interaction between the input targets, indicating a functional relationship between the targets (Fig. 1S). The hub nodes of this PPI network were identified as the most important nodes in the network^32^, which included several ECM components as well as anti-angiogenic factors such as Thbs1 and Thbs2. The expression for a selection of targets from these hub nodes was validated using the HLI model. Concurrent with the downregulation of the three miRNAs, their targets were upregulated, except for Tgfb2 (Fig. 7). However, we we observed a significant dysregulation of Mstn expression after ischaemia (Fig. 2d), which is a highly conserved TGFβ family member that is expressed in skeletal muscle^87^. As Mstn is an inhibitor of muscle growth, it is likely that its downregulation at day 7 post-ischaemia may be needed to enable muscle recovery. We further investigated the roles of these targets by functional enrichment analysis using enrichment mapping^33^. This aimed to understand the pathways and biological functions that may be regulated by this three-miRNA signature in skeletal muscle ischaemia. Here, the over-representation of several “biological themes” was identified. Firstly, the largest biological theme identified was fibrosis. Numerous clusters related to ECM and collagen, PDGF, cell adhesion, and fibroblast proliferation were identified. Fibrosis is a pathological process whereby there is excessive deposition of ECM (in particular, collagen), impairment in ECM degradation, or both of these processes^88,89^. Fibrosis is a hallmark feature of skeletal muscle pathology in CLTI and has been suggested to be a central mechanism of disease progression^13^. Additionally, vascular development and the VSMC cluster may further pertain to the CLTI-associated vascular pathology and associated fibrotic process as there is an ischaemia-induced increase in expression of TGFβ*1* in VSMCs which is associated with fibroblast accumulation and collagen deposition^66,90^.

### Study limitations

There is an inherent limitation of using the HLI model to validate the predicted dysregulation of miRNAs and their targets in CLTI patients, as this model only allows the study of muscle regeneration in response to acute ischaemic injury compared to chronic ischaemia in CLTI patients. We also only validated miRNAs that were enriched in the CLTI vs. non-PAD control group. It is plausible that other miRNAs enriched in the CLTI vs. IC group could also be dysregulated in HLI ischaemic muscles, especially miRNAs from the miR-29 family, including miR-29a and miR-29c. It is also possible that specific miRNAs play different physiological and/or pathological roles in different ischaemic contexts. The presence of DM in individuals with CLTI and IC was not considered in our bioinformatic analysis. It is possible that the diabetic milieu may alter miRNA expression. Further studies will confirm miRNA dysregulation in human muscle from CLTI, IC, and non-PAD controls in the presence and absence of DM. Finally, this study employed a small sample size (*n = 3*) to validate the dysregulated miRNAs from in silico prediction in mice with HLI, and therefore it could be considered a pilot study. Also, a control group with mock procedure would rule out any specific change caused due to the operation procedure itself (the injury from the operation, the anaesthetic, analgetic or antibiotic), and this is something that has not been considered in this study.

## Conclusions

To the best of our knowledge, this is the first study to identify miR-1, miR-133a, and miR-29b as potential regulators of skeletal muscle pathology in patients with CLTI. The results presented here indicate miR-1, miR-133a, and miR-29b as a miRNA signature of skeletal muscle fibrosis in CLTI. Given the enrichment of these miRNAs in transcriptomic data, these miRNAs likely represent central regulators of this muscle pathology and warrant further investigation as potential therapeutics. A defining feature of miRNAs is their pleiotropic nature: a single miRNA can regulate many mRNAs, and a single mRNA can be regulated by many miRNAs^91^. From a therapeutic perspective, this makes miRNAs an attractive candidate for novel ‘systems-based’ therapies that could regulate this broad transcriptomic and pathway dysregulation^19,92^. Each of these miRNAs individually possesses therapeutically relevant properties in this context. However, miRNA-based therapy poses the risk of off-target effects, which must be considered^93^. Considering the potential for off-target effects, it is likely that the translation of miRNA therapeutics will require rational identification and validation of candidate miRNAs along with targeting strategies such as modification of the oligonucleotide to target specific cell types. This has been investigated recently in the case of MRG-229, a miR-29b mimic which was modified with a bicyclic peptide specific for PDGFR-β to be internalised by fibrogenic cells in the lung^65^. Such novel therapeutics may restore homeostasis in the ischaemic limb by targeting pathological transcriptomic dysregulation, restoring it to non-ischaemic levels, and ultimately, preventing amputation and improving patient outcomes. Additionally, there is a distinct possibility to investigate the miR-1, miR-133a, and miR-29b panels as potential combinatorial miRNA therapeutics to target fibrosis. The co-delivery of multiple miRNAs that may co-operatively regulate complementary targets in on-target pathways may allow a lower dose of each individual miRNA to be administered and reduce the likelihood of perturbing off-target pathways^92^. Nevertheless, the roles of these specific miRNAs remain relatively novel in the context of PAD/CLTI and ischaemic skeletal muscle regeneration, and investigation of these miRNAs as potential therapeutics is still warranted.

## Electronic supplementary material

Below is the link to the electronic supplementary material.

Supplementary Material 1

Supplementary Material 2

## Data Availability

RNA-sequencing-based profiles from CLTI, IC and non-PAD adults utilised here can be publicly accessed at https://www.ncbi.nlm.nih.gov/geo/query/acc.cgi?acc=GSE120642. The authors declare that the data supporting the findings of this study are available within the article and its supplementary information files and also available from the corresponding author upon request.
