# Supplementary material for: miR-1, miR-133a, miR-29b and skeletal muscle fibrosis in chronic limb-threatening ischaemia
Source: Sci Rep. 2024 Nov 26;14:29393. doi: 10.1038/s41598-024-76415-9 (PMC11599917; doi:10.1038/s41598-024-76415-9)
Supplement: Supplementary file 2 — Supplementary Material 2 [file 41598_2024_76415_MOESM2_ESM.pdf]

SUPPLEMENTARY INFORMATION

Supplementary Figure S1

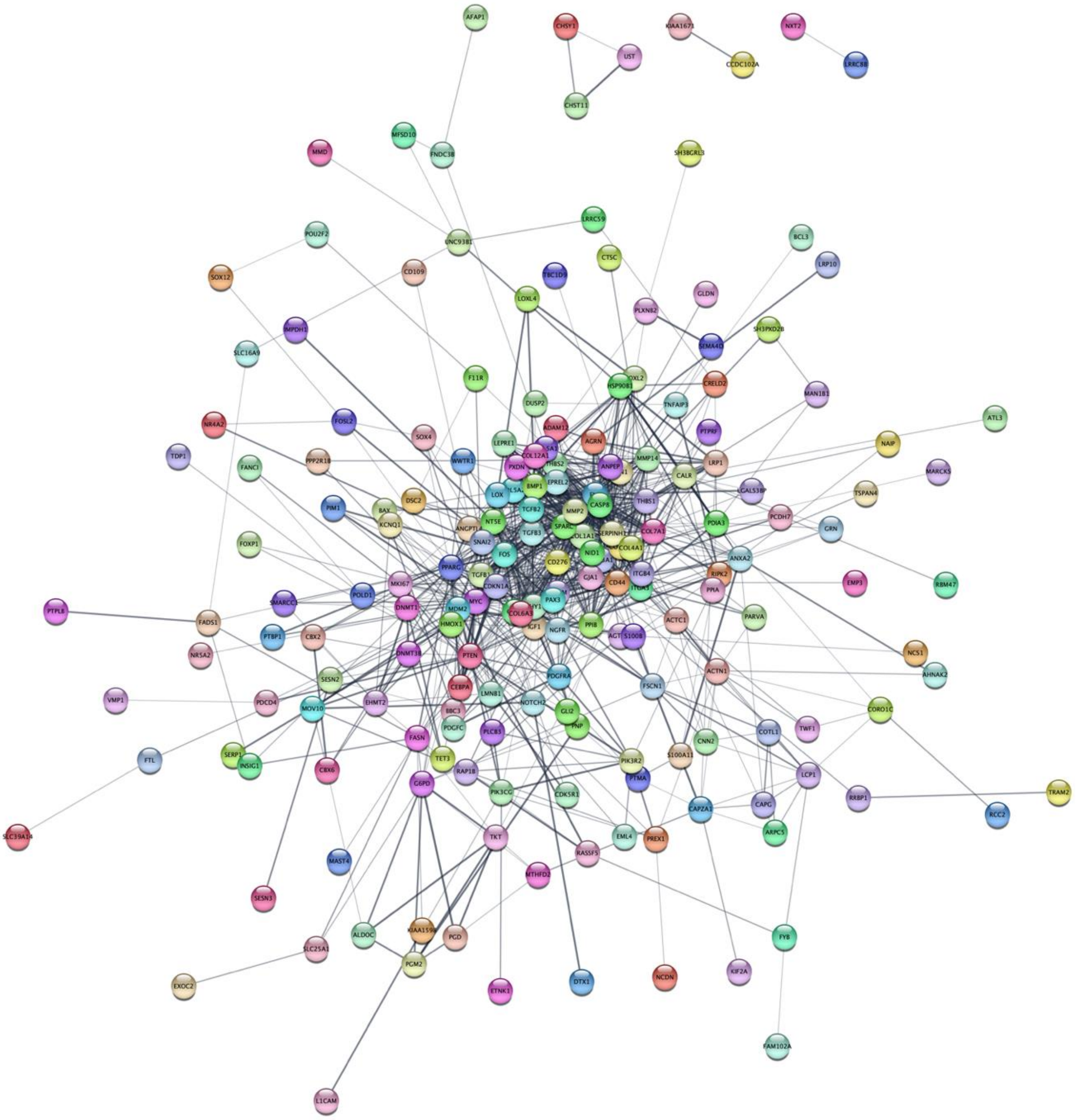

**Number of Nodes:**	235
**Number of Edges:**	965
**Average Node Degree:**	8.21
**Average Local Clustering Coefficient:**	0.46
**Expected Number of Edges:**	5362
**PPI Enrichment p-value:**	< 1.0e-16

Figure S1. PPI network of miR-1, miR-133a, and miR-29b targets upregulated in CLI. A PPI network of the targets of miR-1, miR-133a, and miR-29b that are upregulated in CLI gastrocnemius was constructed using STRING. Nodes represent proteins and edges represent evidence of association between nodes.

SUPPLEMENTARY INFORMATION

Supplementary Figure S2

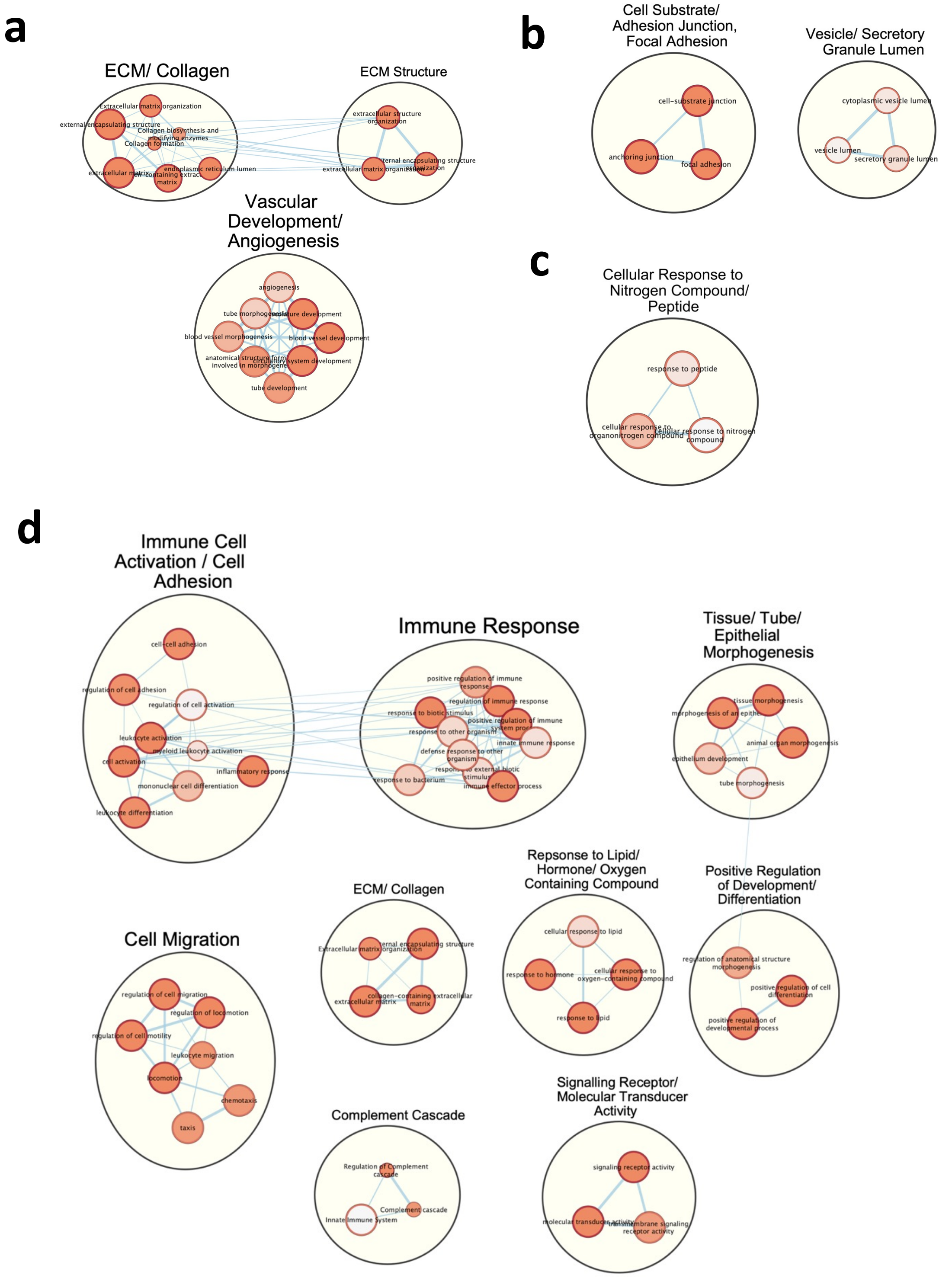

Supplementary Figure S2, continuation

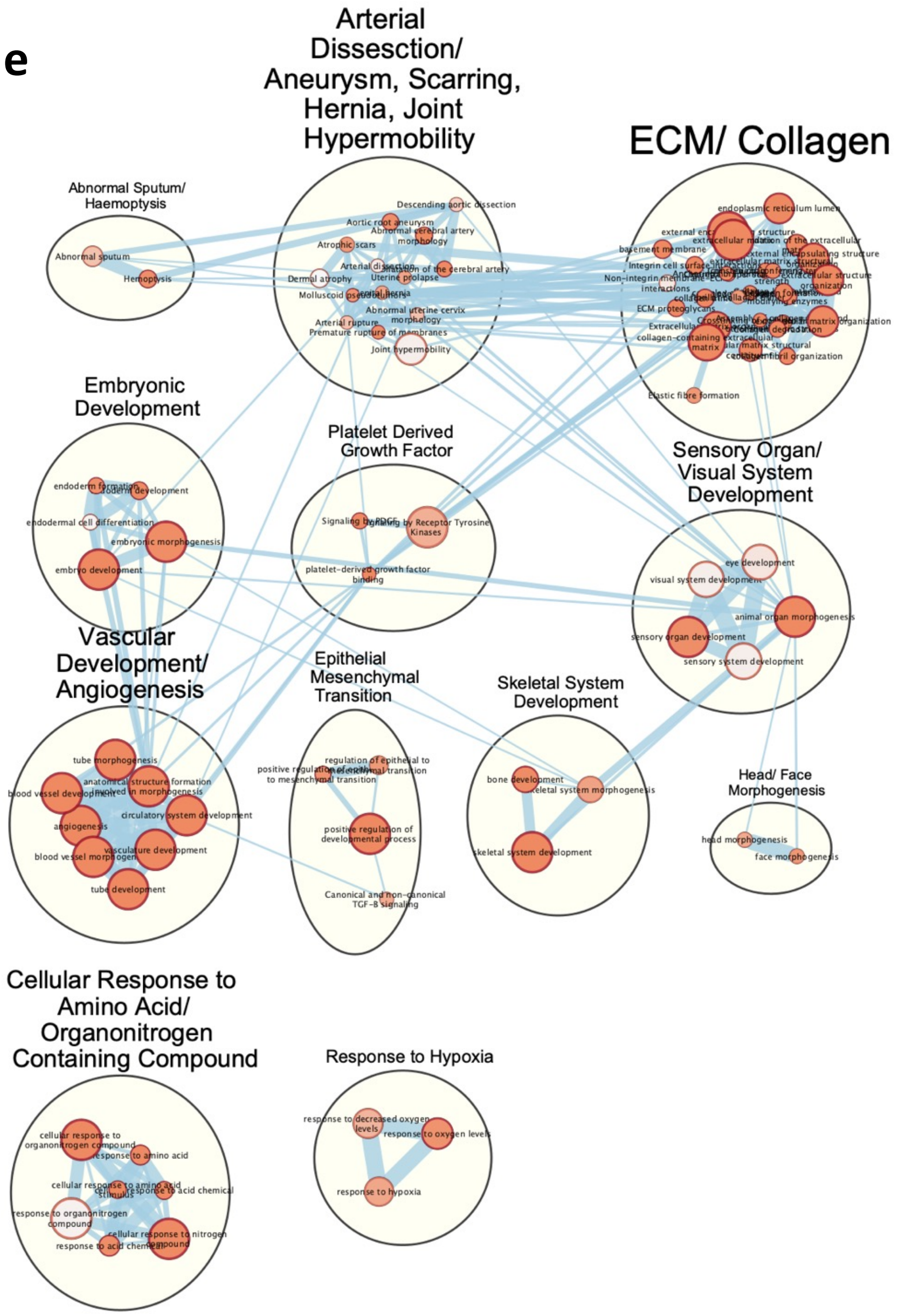

Figure S2. Enrichment Map of the targets of miR-124 (a) miR-1 (b), miR-133a (c) miR-335 (d) and miR-29b (e) which are upregulated in CLTI Muscle. Functional enrichment was performed on the targets of miRNAs that are upregulated in CLTI gastrocnemius using g:Profiler. Enrichment mapping was performed on terms with an FDR <0.05 and term size between 5-1000 using the EnrichmentMap plugin in Cytoscape. Cluster labels created using the AutoAnnotate plugin were manually edited. Nodes represent enriched pathways identified using g:Profiler. Edges represent, and are weighted by, pathway gene set overlap. Node colour is mapped to pathway enrichment significance (orange = lower Q value and white = higher Q value). Node size is mapped to gene set size.

Supplementary Figure S3.

A)

Accession	Mature miRNA ID	Stem Loop	Sequence	Homology
miR-1				
MIMAT0000416 (Human)	hsa-miR-1-3p	hsa-mir-1-2<br>hsa-mir-1-1	UGGAAUGUAAAGAAGUAUGUAU	100%
MIMAT0000123 (Mouse)	mmu-miR-1a-3p	mmu-mir-1a-1<br>mmu-mir-1a-2	UGGAAUGUAAAGAAGUAUGUAU	
miR-133a				
MIMAT0000427 (Human)	hsa-miR-133a-3p	hsa-mir-133a-1<br>hsa-mir-133a-2	UUUGGUCCCCUUCAACCAGCUG	100%
MIMAT0000145 (Mouse)	mmu-miR-133a-3p	mmu-mir-133a-1<br>mmu-mir-133a-2	UUUGGUCCCCUUCAACCAGCUG	
miR-29b				
MIMAT0000100 (Human)	hsa-miR-29b-3p	hsa-mir-29b-1<br>hsa-mir-29b-2	UAGCACCAUUUGAAAUCAGUGUU	100%
MIMAT0000127 (Mouse)	mmu-miR-29b-3p	mmu-mir-29b-1<br>mmu-mir-29b-2	UAGCACCAUUUGAAAUCAGUGUU	
miR-124				
MIMAT0000422 (Human)	hsa-miR-124-3p	hsa-mir-124-1<br>hsa-mir-124-2<br>hsa-mir-124-3	UAAGGCACGCGGUGAAUGCCAA	100% except last two nucleotides
MIMAT0000134 (mouse)	mmu-miR-124-3p	mmu-mir-124-3<br>mmu-mir-124-1<br>mmu-mir-124-2	UAAGGCACGCGGUGAAUGCC	
miR-335				
MIMAT0000765 (Human)	hsa-miR-335-5p	hsa-mir-335	UCAAGAGCAAUAACGAAAAAUGU	100%
MIMAT0000766 (Mouse)	mmu-miR-335-5p	mmu-mir-335	UCAAGAGCAAUAACGAAAAAUGU	
miR-7110				
MIMAT0028117 (Human)	hsa-miR-7110-5p	hsa-mir-7110	UGGGGGUGUGGGGAGAGAGAG	-

B)

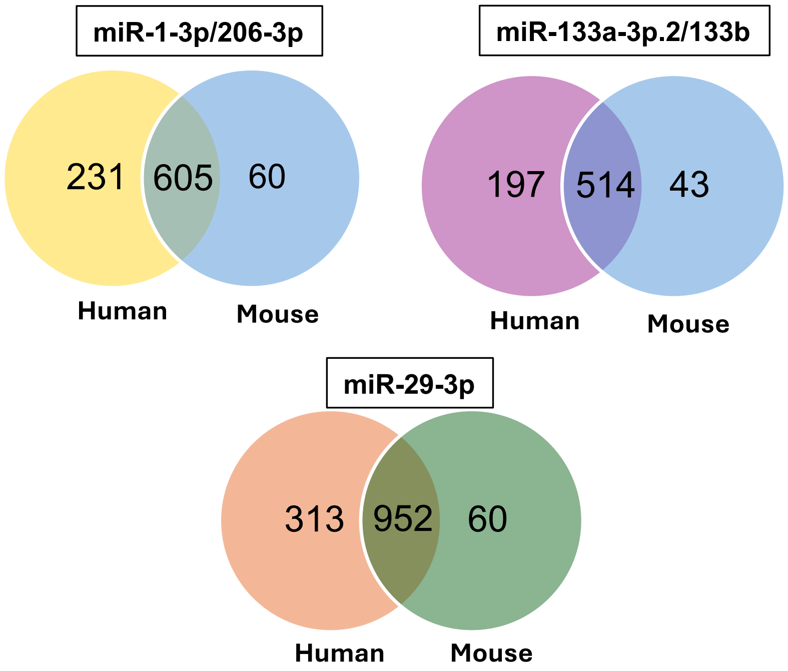

Figure S3. A) miRNA sequence homology in mouse and human. miRNA accession, mature miRNA ID, miRNA stem loop ID(s), and mature miRNA sequences for miR-1(a)-3p, miR-133a-3p, miR-29b-3p, miR124-3p, miR-335-5p and miR-7110-5p in human (*Homo sapiens*, hsa) and mouse (*Mus musculus*, mmu) were obtained from miRBase release 22.1. miR-7110-5p was not annotated in mouse. **B) Venn Diagram showing the number of miRNA-predicted targets conserved in humans and mice.** A list of predicted miRNA protein targets for miR-1/206, miR-133a/miR-133b and miR-29 was obtained using TargetScan.

SUPPLEMENTARY INFORMATION

Major Resources Table

To allow validation and replication of experiments, all essential research materials listed in the Methods should be included in the Major Resources Table below. Authors are encouraged to use public repositories for protocols, data, code, and other materials and provide persistent identifiers and/or links to repositories when available. Authors may add or delete rows as needed.

Animals (in vivo studies)

Species	Vendor or Source	Background Strain	Sex
Mouse	Janvier Labs, France	BALB/c nude	Male

Data & Code Availability

Description	Source / Repository	Persistent ID / URL
GSE120642	NCBI GEO	https://www.ncbi.nlm.nih.gov/geo/query/acc.cgi?acc=GSE120642

List of miRNA Primers

Description	Source / Repository	Persistent ID
miR-27b-3p	Qiagen	YP00205915
hsa-miR-1-3p	Qiagen	YP00204344
hsa-miR-133a-3p	Qiagen	YP00204788
hsa-miR-29b-3p	Qiagen	YP00204679
hsa-miR-124-3p	Qiagen	YP00206026
hsa-miR-335-5p	Qiagen	YP02119293

List of primer sequences for miRNA targets

Gene Name	NCBI Gene ID	Primer Sequence	PrimerBank ID
Rpl13a	22121	F 5' – AGCCTACCAGAAAGTTTGCTTAC<br>R 5' – GCTTCTTCTTCCGATAGTGCATC	334688867c2
Gapdh	14433	F 5' – AGGTCGGTGTGAACGGATTTG<br>R 5' – TGTAGACCATGTAGTTGAGGTCA	6679937a1
Col1a1	12842	F 5' – GCTCCTCTTAGGGGCCACT<br>R 5' – CCACGTCTCACCATTGGGG	34328108a1
Col3a1	12825	F 5' – CCTGGCTCAAATGGCTCAC<br>R 5' – CAGGACTGCCGTTATTCCCG	22832760a1
Col4a1	12826	F 5' – CTGGCACAAAAGGGACGAG<br>R 5' – ACGTGGCCGAGAATTTCACC	33859528a1
Fn1	14268	F 5' – GCTCAGCAAATCGTGCAGC<br>R 5' – CTAGGTAGGTCCGTTCCCACT	26344255a1
Fbn1	14118	F 5' – GGACGCCAATTTGGAGGCT	6679759a1

		R 5' – CTTTCAGCGCATCGTGTCCT	
Sparc	20692	F 5' – GTGGAAATGGGAGAATTTGAGGA<br>R 5' – CTCACACACCTTGCCATGTTT	6678077a1
Mmp2	17390	F 5' – CAAGTTCCCCGGCGATGTC<br>TTCTGGTCAAGGTCACCTGTC	6678902a1
Mmp14	17387	F 5' – CAGTATGGCTACCTACCTCCAG<br>R 5' – GCCTTGCCTGTCACTTGTAAA	31982191a1
Myh7	140781	F 5'ACTGTCAACACTAAGAGGGCA<br>R 5' - TTGGATGATTTGATCTTCCAGGG	18859641a1
Acta1	11459	F 5' – CCCAAAGCTAACCGGGAGAAG<br>R 5' - GACAGCACCGCCTGGATAG	133893192c1
MuRF1 (Trim63)	433766	F 5' – GTGTGAGGTGCCTACTTGCTC<br>R 5' – GCTCAGTCTTCTGTCCTTGGA	21523717a1
Atrogin1 (Fbxo32)	67731	F 5' – CAGCTTCGTGAGCGACCTC<br>R 5' – GGCAGTCGAGAAGTCCAGTC	13385848a1
Myostatin	17700	F 5' – AGTGGATCTAAATGAGGGCAGT<br>R 5' – GTTTCCAGGCGCAGCTTAC	6754752a1
Tgfb2	21808	F 5' - CTTCGACGTGACAGACGCT<br>R 5' – GCAGGGGCAGTGTAAACTTATT	15029686a1
Cd206	17533	F 5' – CTCTGTTCAGCTATTGGACGC<br>R 5' – CGGAATTTCTGGGATTCAGCTTC	6678932a1
Tnfa	21926	F 5' – CCCTCACACTCAGATCATCTTCT<br>R 5' – GCTACGACGTGGGCTACAG	7305585a1
F4/80	13733	F 5' – CCCCAGTGTCCTTACAGAGTG<br>R 5' – GTGCCCAGAGTGGATGTCT	33859546a1
